# Maternal Vitamin B12 Status during Pregnancy and Early Infant Neurodevelopment: The ECLIPSES Study

**DOI:** 10.3390/nu15061529

**Published:** 2023-03-22

**Authors:** Josué Cruz-Rodríguez, Andrés Díaz-López, Josefa Canals-Sans, Victoria Arija

**Affiliations:** 1 Nutrition and Mental Health Research Group (NUTRISAM), Universitat Rovira i Virgili (URV), 43201 Tarragona, Spain; 2 Institut d’Investigació Sanitària Pere Virgili (IISPV), 43005 Tarragona, Spain; 3 Centre de Recerca en Avaluació i Mesura de la Conducta (CRAMC), Department of Psychology, Universitat Rovira i Virgili, 43007 Tarragona, Spain; 4 Institut d’Investigació en Atenció Primària IDIAP Jordi Gol, Institut Català de la Salut (ICS), 08007 Barcelona, Spain; 5 Collaborative Research Group on Lifestyles, Nutrition and Smoking (CENIT), IDIAP Jordi Gol, 43202 Reus, Spain

**Keywords:** vitamin B12 deficiency, pregnancy, prenatal nutrition, infant neurodevelopment, ECLIPSES study

## Abstract

In this prospective cohort study of 434 mother–infant pairs from the ECLIPSES study, we examine the association between maternal vitamin B12 status at the beginning and end of pregnancy and the neurodevelopmental outcomes of infants 40 days after birth in a pregnant population from a Mediterranean region of northern Spain. Maternal vitamin B12 concentrations were determined in the first and third trimesters, and sociodemographic, nutritional, and psychological data were collected. At 40 days postpartum, the Bayley Scales of Infant Development-III (BSID-III, cognitive, language, and motor skills) were administered to the infants and several obstetrical data were recorded. In the multivariable models, medium maternal first-trimester vitamin B12 levels (312 to 408 pg/mL, tertile 2) were associated with better neonatal performance in the motor, gross motor, language, and cognitive skills with respect to tertile 1 (<312 pg/mL). The probability of obtaining a neonatal motor, gross motor, and receptive language score >75th percentile was significantly higher also in the tertile 2 group. In summary, good maternal vitamin B12 status in the early stage of pregnancy appears to be associated with better infant motor, language, and cognitive performance at 40 days postpartum.

## 1. Introduction

Maternal nutrition before and during pregnancy is essential for maintaining normal fetal growth and adequate neurodevelopment because the nutritional supply and nutritional reserves from the mother are the only sources of nutrients for the fetus [1,2].

Several epidemiological studies have reported associations between lower maternal circulating nutrient concentrations during pregnancy and greater neurodevelopmental impairment in infants. Like nutrients such as folate, iron, vitamin D, and polyunsaturated fatty acids [2,3,4], maternal vitamin B12 has a clear effect on the neurodevelopment of children [3,4] and affects a high percentage of the population, even in developed countries. In Europe, prevalence from 18% to 43% has been observed. Moreover, this prevalence is higher in, for example, environments with lower socioeconomic levels [5,6,7,8].

Numerous studies have reported environmental risk factors associated with vitamin B12 deficiency, including diet, smoking, and physical activity [9,10,11,12]. The main causes of vitamin B12 deficiency worldwide are the low consumption of foods of animal origin and absorption problems. It has been reported, for example, that pregnant women who have been strict vegetarians for several years, and even omnivores who consume low amounts of animal foods, are prone to developing vitamin B12 deficiency during pregnancy [9,10,11]. Smoking is another associated factor because vitamin B12 in hydroxycobala-mine form binds to the cyanide present in cigarettes to form a non-toxic compound (cyanocobalamin) that is subsequently eliminated in urine, which thus reduces vitamin B12 reserves [13]. In addition to environmental factors, certain genetic polymorphisms (FUT2, MUT, CUBN, TCN1, and MS4A3) associated with vitamin B12 metabolism have been described, though their impact on the vitamin’s serum levels has not yet been determined [14,15,16]. This hereditary disorder has been related to cardiometabolic, hematological, and neurological alterations [17].

Vitamin B12 is involved in several metabolic mechanisms, and its deficiency in pregnant women can cause hyperglycemia, insulin resistance, obesity, and dyslipidemia, which affect the health of the mother and directly and indirectly influence the development of the baby [18,19]. Moreover, along with folate, vitamin B12 is a required cofactor in one-carbon metabolism, a deficiency of which leads to elevated levels of homocysteine (tHcy) and methylmalonic acid [4,7]. This effect is related to purine and pyrimidine synthesis and, subsequently, to genomic stability [20], thus modifying fetal programming [21]. Elevated pregnancy tHcy causes adverse outcomes in the offspring, ranging from lower scores on expressive language and in gross motor domains [22] to neural-tube defects, whereas moderately elevated preconception tHcy has also been associated with a greater probability of lower psychomotor ability in four-month-old infants [23].

Independently, vitamin B12 plays a key role in intrauterine fetal development, participating in brain growth, myelination, neurogenesis, and synaptic connectivity, mainly in the visual and auditory cortices [4,24]. In the uterus, the fetus is entirely dependent on its mother for nutrition. Fetal exposure to low vitamin B12 (mostly of maternal origin) during certain periods of gestation can therefore have early postnatal consequences for cognitive development, thus affecting memory, language, and visual and auditory processing in the offspring [4,9,24,25]. This latter aspect affects perceptual–motor integration and, consequently, motor skills [4,26].

As far as we know, only seven observational studies have focused on these topics, and their findings are not conclusive. Five studies were conducted in Asian countries [27,28,29,30,31], one was conducted in Canada [32], and one in the Netherlands [33]. Of the five Asian studies, the research conducted by Keskin et al. [27] reported that low maternal vitamin B12 status in the first trimester was associated with impaired motor, language, and social skills in four-month-old infants who were also found to have vitamin B12 deficiency [27]. Two other studies found that low maternal vitamin B12 levels during the third trimester were associated with lower cognition [28,29] and social development [29] in offspring at two years of age. Bhate et al. [30] reported a lower performance on a working-memory task completed by the 9- to 10-year-old children of mothers in the lowest decile of vitamin B12 concentrations during the third trimester of pregnancy. However, children of mothers in the highest vitamin B12 decile performed better on the same task [30]. Vaena et al. [31], on the other hand, found no association with cognitive development in children of the same age [31]. Similarly, in the studies conducted in Canada by Wu et al. [32] in children aged 1.5 years and in the Netherlands by Ars et al. [33] with children aged 6 to 8 years, no evidence was found of an association between maternal vitamin B12 status during the second trimester of pregnancy and offspring neurodevelopment.

In addition, two randomized controlled trials (RCTs) in Asian countries [22,34] have recently reported that children of vitamin B12-supplemented mothers who had high vitamin B12 concentrations in the first [22] and third [22,34] trimesters of pregnancy showed higher scores in expressive language at 2 [34] and 2.5 [22] years of age than children of mothers who did not receive supplements.

It should be borne in mind, however, that all previous observational studies on maternal vitamin B12 status and offspring neurodevelopmental outcomes have evaluated vitamin B12 concentrations only at one point in the pregnancy [27,28,29,30,31,32,33]. Nevertheless, previous findings in this research field suggest that this relationship depends on the pregnancy trimester in which the mother had this deficiency [22,27,28,29,30,31,32,33,34]. The trimester in which the fetus is most susceptible to prenatal vitamin B12 status, and indeed how this may affect child neurodevelopment, therefore remains to be determined. Moreover, it could be said that maternal vitamin B12 status during pregnancy is influenced by multiple factors specific to each population [4,35,36,37]. However, no studies have been conducted among Mediterranean pregnant populations, whose socio-demographic characteristics and Mediterranean-lifestyle traits could promote optimal infant neurodevelopment. Furthermore, since few studies have considered a large range of risk factors simultaneously, the possibility of residual confounding is greater [4,35,36,37]. Further research is therefore needed to better understand these associations.

Since the influence of maternal vitamin B12 status on offspring neurodevelopment has not yet been studied in depth [2,3,4], the aim of this study was to examine the association between maternal vitamin B12 status at the beginning and end of pregnancy and infant neurodevelopment 40 days after birth, adjusting for potential pre-, peri-, and post-natal confounders, in a population of pregnant women from a Mediterranean region of northern Spain (Catalonia).

## 2. Materials and Methods

### 2.1. Study Design and Participants

We conducted a prospective follow-up study to analyze data from pregnant women as well and their children 40 days after delivery. This work is part of the ECLIPSES Study [38]. A total of 791 participants were recruited during the first prenatal visit from 12 sexual- and reproductive-health-care services (ASSIR) of the Catalan Institute of Health (ICS) in the province of Tarragona (Catalonia, Spain) between 2013 and 2017. 

Eligible participants were healthy women over 18 years of age with ≤12 weeks of gestation and without anemia (Hb > 110 g/L). More details of the inclusion/exclusion criteria have been published previously. These include the ability to understand the local and official languages of the region (Spanish or Catalan), and the characteristics of the study. Women were excluded if at the beginning of the study they met any of the following criteria: had had multiple pregnancies, had taken iron supplements in the months before the 12th week of pregnancy (indicated in the ECLIPSES RCT on iron supplementation), or had previously had a severe disease (immunosuppression) or a chronic disease that could affect their nutritional development (cancer, diabetes, malabsorption, or liver disease) [38]. The study was designed in accordance with the Declarations of Helsinki and all procedures were approved by the Ethics Committee of the Institut d’Investigació en Atenció Primaria de Salut (IDIAP) and the Institut d’Investigació Sanitària Pere Virgili (approval ID: 118/2017. Date: 28 September 2017). The ECLIPSES study was registered at www.clinicaltrialsregister.eu with identification number EUCTR-2012-005480-28 and at www.clinicaltrials.gov with identification number NCT03196882. Informed consent forms were signed by all women who participated. 

In addition to the recruitment visit from a midwife before the 12th gestational week (GW), the study involved conducting two more visits during the pregnancy (at weeks 12 and 36) and one at 40 days postpartum. Our analysis includes all women who completed the study up to the evaluation of their child at 40 days postpartum. The sample size was calculated for three groups, with an alpha risk of 0.05, a beta risk of less than 0.2, one-sided contrast, and accordance with sample data. For the motor scale 130 subjects were needed, with a standard deviation (SD) of 11.2 points and a minimum difference to detect from 3 points; for the language scale 129 subjects were needed (SD: 8.2 points and difference to detect from 2.2 points); and for the cognitive scale 125 subjects were needed (SD: 8.8 points and difference to detect from 2.2 points). The women’s serum vitamin B12 levels were tested in the first and third trimesters of pregnancy and the children’s neurodevelopment was assessed at the postpartum visit.

### 2.2. Data Collection

#### 2.2.1. Maternal

##### Sociodemographic Data

The midwives collected information through interview-administered questionnaires on maternal age, educational level, and socio-economic level. Educational level was divided into two groups: low/medium (primary school or secondary studies) and high (university studies or more). Socioeconomic level was classified as low, middle, or high according to the family’s occupational status and using the Catalan classification of occupations (CCO-2011) [39,40].

##### Lifestyle Habits

The Fagerström questionnaire (Fagerström_Q) [41] was used to assess smoking, and the women were classified as smokers or non-smokers. Smoking was evaluated in the three pregnancy trimesters. Women who smoked during at least one trimester were considered smokers, whereas those who did not smoke during the three trimesters of pregnancy were considered non-smokers.

Eating habits were assessed using a self-administered food-frequency questionnaire (FFQ) validated in our population [42]. A nutritionist checked the administered FFQs for completeness or discrepancies and clarified portion sizes. The FFQ consisted of 45 items categorized into food groups. The size and weight of a serving portion were standardized according to the validation questionnaire [42]. With this information we used the French food-composition table (REGAL—Répertoire Général des Aliments) [43] to extract daily energy intake (kcal/day), macronutrients (g/day), and micronutrients (mg or µg/day), since this food-composition table is closest to the characteristics of our population from our own analysis and was used for 99% of the food consumed. When this table did not contain any food because it was typical of the area, it was complemented by the Spanish food-composition table [44]. 

The FFQ was administered in the three trimesters of gestation, and a mean value was calculated to represent intake during gestation. We were interested in the adherence to the Mediterranean diet, which was assessed using the relative Mediterranean-diet (MedDiet) score based on the intake of nine components (fruit, vegetables, legumes, cereals, fresh fish, olive oil, meat, dairy foods, and alcoholic drinks) [45]. The score ranges from 0 to 18 points, with larger values indicating greater adherence to the MedDiet and therefore a diet of higher quality. The MedDiet was recorded in the three trimesters of gestation, and a mean value was calculated to represent the MedDiet during gestation. Since there are no pre-established cutoff points for the pregnant population, the MedDiet score was divided into tertiles. Alcohol consumption during pregnancy was assessed as yes or no [45]. Physical activity (PA) was measured using the short version of the International Physical Activity Questionnaire (IPAQ-S) [46]. We recorded the type of PA (walking, moderately intense PA, and vigorous PA), frequency (number of times/week), and duration (minutes/day). To determine the metabolic equivalents (METs/minutes/week) for each type of PA, we averaged the usual frequency and duration (minutes/week) and multiplied them by a constant according to their energy expenditure (walking: 3.3 METs; moderately intense PA: 4.0 METs; and vigorous PA: 8.0 METs) [46]. Total PA was obtained by adding the METs/min/week for each type of PA. PA was recorded in the three trimesters of gestation, and a mean value was calculated to represent PA during gestation and divided into tertiles for our analysis.

##### Anthropometric Measurements

Maternal weight (in kg to the nearest 0.1 kg) and height (in cm to the nearest 0.1 cm) were measured and body mass index (BMI) was calculated from these measurements (weight (kg)/height (m)^2^). Based on WHO criteria [47], the women were classified as normal weight (BMI < 25 kg/m^2^) or overweight/obese (BMI ≥ 25 kg/m^2^) in the first trimester. Total gestational weight gain (GWG) was the difference between the weights measured at the first- and third-trimester visits.

##### Psychological Data

At the first- and third-trimester visits, the State-Trait Anxiety Inventory (STAI) [48] was applied to determine the mothers’ emotional state. This questionnaire measures the levels of trait anxiety (20 items: stable and dispositional anxiety) and state anxiety (20 items: situational and transient anxiety). For this study, we used only the state-anxiety scores. At 40 days postpartum the mothers also completed the Parental Stress Index Short Form 4th edition (PSI-4-SF) [49]. This consists of 36 statements to measure stress directly associated with parenting. For this study, we used only the total score for dysfunctional parent–child interaction, which is related to mother–child attachment.

##### Biochemical Data

Venous blood samples were taken at weeks 12 and 36 of gestation. The serum was separated by centrifugation and stored in aliquots at −80 °C in the Biobank of the reference hospital until processing. Serum vitamin B12 was determined with the ADVIA Centaur Vitamin B12 immunoassay method. Vitamin B12 levels (pg/mL) were divided into tertiles, with the pregnant woman categorized as having low, medium, or high vitamin B12 levels (tertile 1, tertile 2, and tertile 3, respectively). Red-blood-cell folate (RBC folate) was also determined with an ADVIA Centaur immunoassay.

#### 2.2.2. Infant

##### Obstetrical and Birth Data

During the visit conducted 40 days after delivery, data on the infants’ sex, weight, length, head circumference at birth, and gestational age at birth were collected from their health cards. The mothers were also asked about which type of feeding they used.

##### Psychological Data

At the 40-days-postpartum visit, two trained psychologists applied the Bayley Scales of Child Development (BSID-III) [50], which assesses neurodevelopment in children aged 0 to 42 months. These comprise three general scales: cognitive, motor, and language. The language scale comprises two subscales assessing receptive and expressive language, whereas the motor scale comprises two subscales that assess fine and gross motor skills. The results of each scale are expressed in composite scores with a mean of 100 and a standard deviation of 15, except for the scores of the receptive- and expressive-language subscales and fine and gross motor skills, which are expressed in scalar scores with a mean of 10 and a standard deviation of 3.

### 2.3. Statistical Analysis

Descriptive data were expressed as means or geometric means (±SD) for continuous variables and as a number (percentage) for qualitative variables. The Shapiro–Wilk test and visual inspection (quantile–quantile plot) were used to test continuous data for normality. Due to the left-skewed distribution, fine motor and cognitive scales were log-transformed prior to analysis. For descriptive statistics, the variables are expressed as geometric means (±SD) by transforming the mean of the logarithmic value to the original scale, whereas for linear-regression analysis, estimates are log-transformed data. 

The women were classified into tertiles of serum vitamin B12 concentrations, and the lowest tertile was used as the reference category. Differences in the child cognitive-development scales between maternal vitamin B12 tertiles were compared using the ANOVA test.

Associations between maternal vitamin B12 levels (in tertiles) in the first and third trimesters of pregnancy (independent variable) and each infant neurodevelopment scale (cognitive, language, and motor skills, all continuous variables) were assessed by separate multivariable linear-regression models using the ENTER method adjusted for the following possible confounding variables based on previous knowledge: maternal age (years), BMI (0: <25 kg/m^2^ (reference), 1: ≥25 kg/m^2^), gestational weight gain (kg), educational level (0: primary/secondary (reference), 1: university), social class (0: low/medium (reference), 1: high), smoking during pregnancy (0: no (reference), 1: yes), previous parity (0: no (reference), 1: yes), physical activity during pregnancy (METS/min/week, tertiles (T1: (reference)), total energy intake (kcal/day), MedDiet (score), vitamin B12 intake (µg), folate intake (µg), RBC folate levels (nmol/L), PSI, mother–child interaction (score), anxiety state (score), sex of child (0: male (reference), 1: female), gestational age at birth (weeks), type of lactation (0: breastfeeding (reference), 1: formula/mixed), neonatal weight–height ratio (g/m), and birth head circumference (cm). The following confounders had missing values: physical activity (7.6%); dietary covariates, including the MedDiet score, energy intake, vitamin B12 intake, and folate intake (all 3.2%); Parenting Stress Index, (3.9%), mother’s anxiety state in the first trimester (9.6%) and third trimester (11.9%); RBC folate levels (15.4%); and birth head circumference (7.3%). To prevent bias associated with covariates with missing values, we adopted multiple imputation with the chained-equations method to impute missing data [51] based on correlating missing variables with other participant characteristics (maternal age and BMI). For each analysis, we created 20 imputed data sets and pooled the results using the MI command in Stata. Estimates were presented as a β coefficient (β) and 95% confidence intervals (CIs). All these maternal and child covariates were selected a priori as potential confounders based on previously reported/known associations/risk factors for offspring neurodevelopment [9,10,11,12]. We assessed multicollinearity by inspecting the tolerance (1/VIF) values and variance-inflation factors (VIFs) for this multivariable model. All tolerance values were greater than 0.7 and all VIFs were less than 2.0 [52], which suggests there were no concerns with multicollinearity. Therefore, none of the covariates were removed from the models. 

Each neurodevelopmental scale was also subsequently assessed as a binary outcome (≤75th percentile (reference) and >75th percentile). Separate multivariable logistic-regression models were similarly applied to examine the odds (OR, 95% CIs) of being >75th percentile for each individual neurodevelopmental scale in 40-day-old children in relation to maternal vitamin B12 levels (in tertiles) in the first and third trimesters of pregnancy. Statistical significance was set at *p*  <  0.05. The statistical analysis was performed using STATA software version 15.0 (StataCorp LP, Collage Station, TX, USA).

## 3. Results

### 3.1. Characteristics of Study Participants 

A total of 434 mother–child pairs (50.9% boys and 49.1% girls) was evaluated. Table 1 shows the sociodemographic, lifestyle, and psychological characteristics and percentage of vitamin B12 deficiency of the mothers and the BSID-III scores of the children 40 days after birth. 

Overall, the mean age of the mothers was 30.8 ± 5.0 years, with a mean first-trimester BMI of 24.8 ± 4.3 kg/m^2^ and a mean gestational weight gain of 10.3 ± 3.6 kg. According to the Institute of Medicine (IOM) recommendations, 39% of the mothers satisfied the criteria for GWG, whereas 41% fell below them and 20% exceeded them. A third (36%) of the women had a university education, 18.4% were from a high social class, and 14.5% were smokers during pregnancy. The mean physical activity score was 2362.8 (2473.7) METs/min/week and the mean MedDiet score was 9.7 (2.1). The mean vitamin B12 intake was 4.3 (1.2) µg (Table 1). There were no significant differences in most baseline characteristics between the pregnant women who were included in the analysis and those who were not (Supplementary Table S1).

The anthropometric measurements of the babies at birth were normal (mean weight = 3284.7 ± 463.0 g, mean length = 49.2 ± 2.1, and mean head circumference = 34.5 ± 1.5). The mean gestational age was 39.6 ± 2.2 weeks and 72.6% of the mothers breastfed their babies during their first 40 days of life (Table 1).

The BSID-III scores of children according to maternal vitamin B12 tertiles during the first and third trimesters showed no statistically significant differences. However, the children of mothers with medium vitamin B12 levels (312 to 408 pg/mL, tertile 2) during the first trimester scored higher on this scale than those of mothers in the lowest tertile (<312 pg/mL) (Table 2).

### 3.2. Associations of Maternal Vitamin B12 Levels with BSID-III Scores 

Multiple linear-regression models adjusted for various environmental factors show that medium maternal vitamin B12 levels during the first trimester of pregnancy (312 to 408 pg/mL, tertile 2) were associated with higher BSID-III scores for motor scale (β = 2.766, 95% CI = 0.029, 5.504; *p* = 0.048), gross motor scale (β = 0.706, 95% CI = 0.153, 1.260; *p* = 0.012), language scale (β = 2.199, 95% CI = 0.191, 4.207; *p* = 0.032), and cognitive development (β = 0.267, 95% CI = 0.005, 0.048; *p* = 0.017). These models also demonstrated a positive effect on several BSID-III scores for educational level, physical activity, smoking habit, dietary intake of vitamin B12, and neonatal weight–length ratio (Table 3). No other significant associations were observed for the motor scale in the first trimester. Similarly, no significant associations were observed between serum vitamin B12 levels and BSID-III scores in the third trimester (Table 3).

### 3.3. Maternal Vitamin B12 Levels and the Risk of Scoring >75th Percentile on the BSID-III Scores

**Figure 1:**
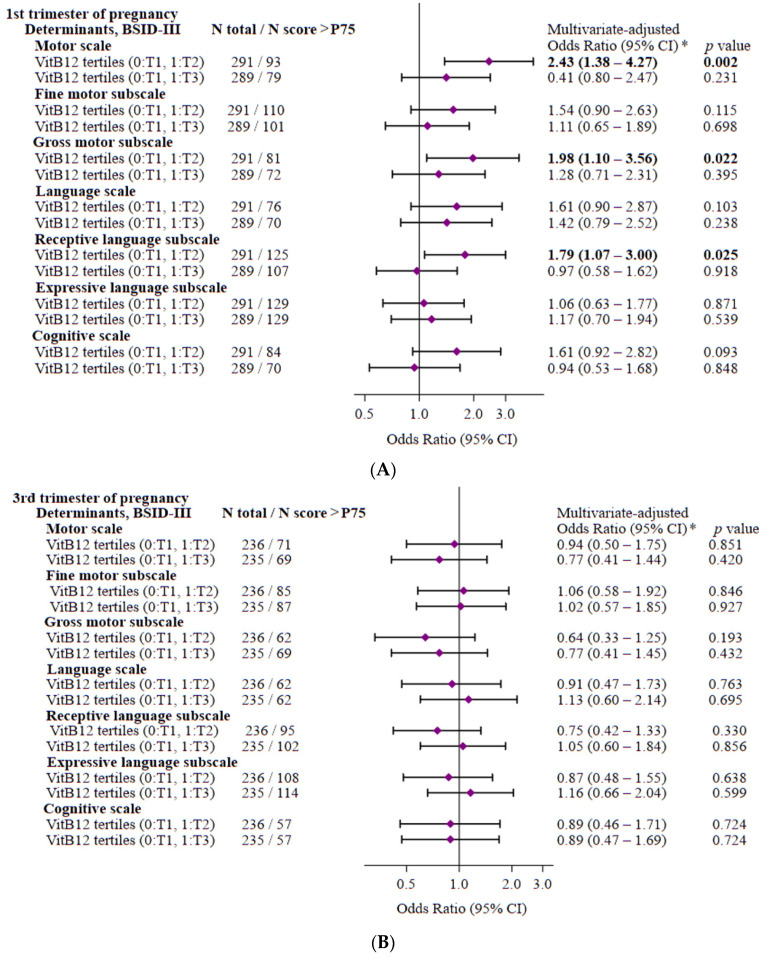
Probability of having scores above the 75th percentile on the Bayley Scales of Child Development (BSID-III) at 40 days after birth in children according to tertiles of maternal vitamin B12 concentrations during the first (**A**) and third (**B**) trimester of pregnancy. * Models of multiple logistic regression were performed, adjusting for the following variables: vitamin B12 tertiles at 1st trimester (T1 (*n* = 146), reference: <312 pg/mL (<230 pmol/L), T2 (*n* = 145): 312–408 pg/mL (230–301.1 pmol/L), and T3 (*n* = 143): ≥ 409 pg/mL (≥301.8 pmol/L)) and vitamin B12 tertiles at 3rd trimester (T1 (*n* = 118), reference: <232 pg/mL (<171.2 pmol/L), T2 (*n* = 118): 232–318 pg/mL (171.2–234.7 pmol/L), and T3 (*n* = 117): ≥319 pg/mL (≥235.4 pmol/L)) depending on the main exposure, maternal age (years), BMI (0: <25 kg/m^2^, 1: ≥25 kg/m^2^), gestational weight gain (kg), educational level (0: primary/secondary, 1: university), social class (low/medium, high), smoking (0: no, 1: yes), previous parity (0: no, 1: yes), physical activity (METS/min/week, tertiles), total energy intake (kcal/day), adherence to the Mediterranean diet (score), vitamin B12 intake (µg), folate intake (µg), RBC folate levels (nmol/L), Parenting Stress Index (score), mother’s anxiety state 1st trimester (score), mother’s anxiety state 3rd trimester (score), sex of child (0: male, 1: female), gestational age at birth (weeks), type of lactation (0: breastfeeding, 1: formula/mixed), neonatal weight–length ratio (g/m), and birth head circumference (cm). The diamonds represent the odds ratio (OR) and the whisker plots represent 95% CIs. *p*-values in bold type are statistically significant.

To evaluate what appeared to be better neurodevelopment, we therefore divided the children into quartiles according to their Bayley III assessment and, taking into account statistical power, used the top 75th percentile of the study sample to define elevated neurodevelopment. According to the BSID-III scores from the first trimester of pregnancy, above the 75th percentile were 27.2% of the babies on the cognitive scale, 26.3% of the babies on the language scale, 40.8% on the receptive-language subscale, 54.8% on the expressive-language subscale, 31.4% on the motor scale, 37.1% on the fine motor subscale, and 27.5% on the gross motor subscale. Multiple logistic-regression models showed that the children of mothers with medium vitamin B12 levels (tertile 2) during the first trimester of pregnancy were more likely to score >75th percentile on the motor scale (OR = 2.43, 95% CI = 1.38, 4.27, *p*  = 0.002), and, more specifically, on the gross motor subscale (OR = 1.98, 95% CI = 1.104, 3.56, *p*  = 0.022) (Figure 1), than the children of mothers with lower vitamin B12 levels (tertile 1). Similarly, the risk of scoring >75th percentile on the receptive-language subscale (OR = 1.79, 95% CI = 1.07, 3.00, *p*  = 0.025) was significantly higher in the children of pregnant women who had medium vitamin B12 levels in the first trimester of pregnancy. In the third trimester, no significant associations were observed (Figure 1).

## 4. Discussion

In this study we found that medium vitamin B12 levels at the beginning of pregnancy in healthy pregnant women from the Spanish Mediterranean area affected the neurodevelopment of their children at 40 days postpartum in the motor area, gross motor skills, and language and cognitive development. Although the BSID-III scores of children were within the normal range, a sufficient maternal vitamin B12 status was associated with a greater probability of children having better motor, gross motor, and receptive-language skills (>75th percentile). 

In the present study, we analyzed the relationship between B12 and neurodevelopment using tertiles of the B12 levels because only very few pregnant women were below the B12-deficiency cutoff level defined by the WHO (200 pg/mL) [53]. Our lowest tertile (<312 pg/mL) was therefore similar to the WHO’s marginal-deficiency value (200 to 300 pg/mL) [53]. We found an association between B12 marginal-deficiency (tertile 1) values and lower scores on the motor, language, and cognitive scales, whereas a slighter, insignificant improvement was observed in tertile 3. This suggests that the effect of maternal vitamin B12 on the infant’s neurodevelopment is not completely linear.

The findings of scarce studies with regard to maternal vitamin B12 status during pregnancy and its effects on child neurodevelopment [27,28,29,30,31,32,33] have been heterogeneous. The methodological characteristics of the studies, including the different design and B12-deficiency cutoff values, as well as the socio-demographic factors of each population, may be behind this diversity in results. Those conduced in developing Asian countries [27,28,29] reported a negative effect on neurodevelopment; however, neither of the two studies realized in developed countries, such as Canada [32] and the Netherlands [33], found any effect of low B12 levels. 

With regard to age in evaluations of infant neurodevelopment, only a recent prospective cohort study by Keskin et al. [27] in Turkey was conducted at a very early age, specifically, four months (n = 88), though only a developmental-screening test (Denver test) was used. In support of our results, the above study found that mothers with vitamin B12 deficiency during the first trimester had babies with lower scores on the development test [27]. However, unlike our study, the authors did not provide specific data on motor skills, cognitive development, and language. 

The relationship found in our study between low B12 values and motor scales was observed only on the gross motor subscale. It should be borne in mind that the fine motor skills evaluated by the BSID-III scale at 40 days, such as the infant’s prehension, perceptual–motor integration, visual object tracking, and response to tactile information, are precise outcomes that depend on many interconnected neurophysiological factors that are still maturing [26,54,55]. However, the gross motor subscale assesses skills that require muscle groups and some degree of physical effort (infant’s movement of limbs and torso, static positioning, and balance) that are more related to the prenatal maturation of the CNS and may be more easily observed at this neonatal age (40 days) [26,55]. However, the gross motor subscale assesses skills that require muscle groups and some degree of physical effort (the infant’s movement of limbs and torso, static positioning, and balance) that are more related to the prenatal maturation of the CNS and may be more easily observed at this neonatal age (40 days) [4,35]. It is important to note that in the first two years of human life, gross motor development is the most important indicator of wellbeing and general development and is therefore of great importance for early developmental screening [56]. Early gross motor skills facilitate the later psychological development of the child in several areas, such as fine motor skills and cognitive and socio-emotional development [56,57].

On the other hand, considering the reported effect of vitamin B12 on the maturation of the visual and auditory cortex [4,24], our results show a significant relationship between vitamin B12 level and receptive language at 40 days. At this age, although the assessment of language development is still quite subtle [58], our data, based on the discrimination of sounds (collected by BSID-III), support the effect of B12 on prenatal brain maturation. In contrast, none of the other previous observational studies [28,29,30,31,32,33] found an association between language in children from two years of age or when maternal vitamin B12 levels were evaluated in the second or third trimester of pregnancy. It is likely that at this age the effect of B12 deficiency on language is offset by stimulation from the postnatal environment or is related to the later period of pregnancy. However, RCTs [22,34] reported that the children of mothers supplemented with and presenting higher serum concentrations of vitamin B12 during the first [22] and third [22,34] trimesters of pregnancy had higher expressive-language scores at 2 [34] and 2.5 [22] years of age than the children of mothers who did not receive supplements. 

In the present study, we found an association between maternal serum vitamin B12 levels in early or late pregnancy and cognitive development. Other studies observed a similar effect in 2-year-olds from Singapore [28] and 2- [29] and 9year-olds [30] from India in the third trimester, though they were also deficient in other micronutrients, such as vitamin B6, [28] folate, and iron [29,30], that are important to the neurodevelopment of children [59]. However, in our study, besides the fact that maternal folate deficiency was very low (3.2% in the first trimester), our multivariate analysis, in which several serum micronutrients were controlled, meant that we were able to attribute the effect of vitamin B12 deficiency regardless of the other deficiencies.

Similar to other authors, we found that neurodevelopment was influenced by other factors, such as the dietary intake of vitamin B12 [22,28,34,60], socioeconomic and educational level [30,61,62,63], physical activity, smoking [64,65], neonatal weight–length ratio, and head circumference [29,30,31,33]. We also observed that a higher maternal dietary intake of vitamin B12 improved certain infant neurocognitive abilities, regardless of vitamin B12 levels. This may be because serum vitamin B12 levels are a late marker of intake and therefore do not reflect the current intake [66,67], as was reported in a systematic review with dose–response meta-analyses of vitamin B12 intake and biomarkers [68] and in a previous study by our research group [12].

Physical activity during pregnancy can promote neurogenesis, proliferation, and neuronal plasticity in children through the action of various (psychological, hormonal, respiratory) mechanisms [69].

It has been reported that intrauterine exposure to tobacco can generate cognitive improvements in response to greater cholinergic activation and cortical arousal derived from nicotine stimulation [64,65,70]. Many studies have observed an association between fetal-growth restriction and poorer neurodevelopmental outcomes [71,72].

Previous studies have assessed maternal vitamin B12 concentrations at the beginning [27] and end [28,29,30,31] of pregnancy. However, we only found effects of vitamin B12 deficiency in the first trimester of pregnancy, even though vitamin B12 levels were lower in the final period of gestation. This suggests that the alteration in neurodevelopment has more to do with the time in which fetal development is more vulnerable to vitamin B12 deficiency than with the serum levels of the vitamin. Indeed, vitamin B12 participates in a wide range of critical processes that are important to the development of the CNS, and some of these (such as neurogenesis) occur mainly in the first trimester of pregnancy, whereas others (such as myelination and synaptogenesis) are more transversal in fetal development [4,24]. Therefore, we assume that studies [28,29,30] that observed an effect of maternal vitamin B12 deficiency on neurodevelopment during the third trimester of pregnancy would also have observed this effect during the first. This indicates the importance of ensuring adequate serum levels of vitamin B12 even before conception.

This study has several limitations. Despite our efforts, participation at 40 days postpartum was lower than at initial evaluation during the first trimester. Another limitation was the lack of additional vitamin B12 biomarkers, such as homocysteine, to improve the evaluation of vitamin status. Since the determination of serum vitamin B12 reflects late deficiency, its combination with another marker such as methylmalonic acid or holotranscobalamin, which are early markers of deficiency, could help to broaden the evaluation of vitamin status [66,67]. Moreover, we did not assess genetic variants related to vitamin B12 metabolism. Nor did we measure serum vitamin B12 levels in the infants. However, as this very early stage the infants depend almost exclusively on the vitamin B12 status of their mothers during gestation and lactation [37]. Moreover, although the infants were assessed at a very early age, administration of the Bayley scales (a validated instrument used internationally to assess infants), allied to the relatively large sample size, enabled us to find significant results.

The strengths of this study lie In the fact that, to the best of our knowledge, it is the first to assess the effect of maternal vitamin B12 levels at two stages of pregnancy on neurodevelopment at 40 days postpartum while adjusting for a wide range of confounding factors, such as diet and maternal psychological aspects. Moreover, different ethnic groups, social classes, and educational levels were represented in the sample and the data were collected using validated questionnaires and standardized techniques.

## 5. Conclusions

In a sample of healthy women from a Spanish Mediterranean region, a sufficient maternal vitamin B12 status in the first trimester of pregnancy, adjusted for various environmental and lifestyle factors, was associated with better motor, language, and cognitive performance in their offspring 40 days after birth. Our findings support the need to assess vitamin B12 levels from the beginning of pregnancy to promote an adequate course of pregnancy and ensure optimal infant neurodevelopment. 

## Figures and Tables

**Table 1:** Descriptive data of the mother and offspring: sociodemographic data, health habits, nutrition, and psychological aspects (*n* = 434).

Maternal Characteristics	Summary Statistics
Age (years) ^#^	30.8 ± 5.0
BMI initial (kg/m^2^) ^#^	24.8 ± 4.3
Gestational weight gain (kg) ^#^	10.3 ± 3.6
Educational level, *n* (%)	
Low (primary/secondary)	278 (64.1)
High (university)	156 (35.9)
Social class, *n* (%)	
Low/medium	354 (81.6)
High	80 (18.4)
Smoking during pregnancy, *n* (%)	
No	371 (85.5)
Yes	63 (14.5)
Alcohol consumption during pregnancy, *n* (%)	
No	363 (86.4)
Yes	57 (13.6)
Physical activity during pregnancy (METs/min/week) ^#^	2362.8 ± 2473.7
Tertile 1 (METs/min/week)	730.4 ± 626.5
Tertile 2 (METs/min/week)	1578.3 ± 289.8
Tertile 3 (METs/min/week)	4801.0 ± 3007.5
MedDiet during pregnancy (score) ^#^	9.7 ± 2.1
Energy intake during pregnancy (kcal) ^#^	2087.1 ± 470.3
Vitamin B12 intake during pregnancy (µg) ^#^	4.3 ± 1.2
Folate intake during pregnancy (µg) ^#^	199.9 ± 59.3
Previous parity, *n* (%)	
No	190 (43.8)
Yes	244 (56.2)
Parenting Stress Index ^#^	50.6 ± 7.9
Mother’s anxiety state 1st trimester (score) ^#^	17.3 ± 8.5
Mother’s anxiety state 3rd trimester (score) ^#^	19.2 ± 8.7
Vitamin B12 levels 1st trimester (pg/mL) ^#^	374.2 ± 127.7
Marginal vitamin B12 deficiency (200 to <300 pg/mL), *n* (%)	115 (26.5)
Vitamin B12 deficiency (<200 pg/mL), n (%)	14 (3.2)
Vitamin B12 levels 3rd trimester (pg/mL) ^# +^	305.2 ± 138.0
Marginal vitamin B12 deficiency (200 to <300 pg/mL), *n* (%)	154 (43.6)
Vitamin B12 deficiency (<200 pg/mL), *n* (%)	62 (17.5)
RBC folate levels (nmol/L)	570.4 ± 207.3
Baby Characteristics	
Sex, *n* (%)	
Male	221 (50.9)
Female	213 (49.1)
Gestational age at delivery (weeks) ^+^	39.6 ± 2.2
Type of feeding, *n* (%)	
Breastfeeding	315 (72.6)
Mixed feeding/infant formula	119 (27.4)
Birth weight (g) ^+^	3284.7 ± 463.0
Birth height (cm) ^+^	49.2 ± 2.1
Birth head circumference (cm) ^+^	34.5 ± 1.5
Weight–length ratio neonatal (g/m) ^+^	66.4 ± 7.6
BSID-III at 40 days	
Motor scale (score) ^+^	107.5 ± 11.2
Fine motor (score) ^+^ *	11.3 ± 1.2
Gross motor (score) ^+^	11.8 ± 2.3
Language scale (score) ^+^	96.2 ± 8.2
Receptive language (score) ^+^	10.6 ± 2.1
Expressive language (score) ^+^	8.0 ± 1.5
Cognitive scale (score) ^+^ *	101.3 ± 1.1

Values are expressed as a mean ± SD (standard deviation) ^#^ or n = number (%). Abbreviations: BMI, body mass index; METs, metabolic equivalent of task; MedDiet, adherence to the Mediterranean diet; RBC folate, red-blood-cell folate; BSID-III, Bayley Scales of Infant Development III. ^+^ *n* = 353. * Fine motor and cognitive scales were not normally distributed and therefore log-transformed; data are geometric mean ± SD. Vitamin B12 equivalencies: 200 pg/mL = 150 pmol/L, 300 pg/mL = 220 pmol/L. Missing value: PA during pregnancy (*n* = 33 (7.6%)); MedDiet (*n* = 14 (3.2%)); energy intake during pregnancy (*n* = 14 (3.2%)); vitamin B12 intake during pregnancy (*n* = 14 (3.2%)); folate intake during pregnancy (*n* = 14 (3.2%)); Parenting Stress Index (*n* = 17 (3.9%)); mother’s anxiety state 1stT (*n* = 42 (9.6%)); mother’s anxiety state 3rdT (*n* = 52 (11.9%)); RBC folate levels (*n* = 67 (15.4%)); birth head circumference (*n* = 32 (7.3%)).

**Table 2:** Means of the Bayley Scales of Infant Development III (BSID-III) scores at 40 days after birth according to tertiles of maternal vitamin B12 concentrations in the first (*n* = 434) and third trimester of pregnancy (*n* = 353).

BSID-III	Vitamin B12 in the 1st Trimester				Vitamin B12 in the 3rd Trimester			
Determinants	Tertile 1 (<312 pg/mL) (*n* = 146)	Tertile 2 (312–408 pg/mL) (*n* = 145)	Tertile 3 (≥409 pg/mL) (*n* = 143)	*p* Value	Tertile 1 (<232 pg/mL) (*n* = 118)	Tertile 2 (232–318 pg/mL) (*n* = 118)	Tertile 3 (≥319 pg/mL) (*n* = 117)	*p* Value
	Mean ± SD	Mean ± SD	Mean ± SD		Mean ± SD	Mean ± SD	Mean ± SD	
Motor scale	106.9 ± 10.0	108.8 ± 13.2	107.7 ± 10.7	0.340	107.1 ± 13.8	107.1 ± 10.4	107.5 ± 10.0	0.948
Fine motor subscale *	11.2 ± 1.2	11.6 ± 1.2	11.2 ± 1.2	0.219	11.2 ± 1.2	11.4 ± 1.2	11.1 ± 1.2	0.655
Gross motor subscale	10.8 ± 2.2	11.4 ± 2.3	11.1 ± 2.3	0.162	11.1 ± 2.4	10.8 ± 2.3	11.1 ± 2.1	0.388
Language scale	95.1 ± 7.9	97.2 ± 8.3	96.0 ± 8.6	0.095	96.2 ± 8.0	96.1 ± 8.9	96.7 ± 7.9	0.830
Expressive-language subscale	7.8 ± 1.4	8.1 ± 1.6	8.1 ± 1.6	0.219	8.0 ± 1.3	8.1 ± 1.7	8.1 ± 1.5	0.860
Receptive-language subscale	10.4 ± 2.1	10.8 ± 2.0	10.4 ± 2.0	0.110	10.5 ± 2.2	10.4 ± 2.2	10.7 ± 1.9	0.574
Cognitive scale *	100.5 ± 1.1	103.0 ± 1.1	100.9 ± 1.1	0.051	100.8 ± 1.1	101.4 ± 1.1	102.0 ± 1.1	0.564

Values are expressed in means ± SD (standard deviation). * Fine motor and cognitive scales were not normally distributed and therefore log-transformed; data are geometric mean ± SD. Vitamin B12 equivalencies: 200 pg/mL = 150 pmol/L, 300 pg/mL = 220 pmol/L.

**Table 3:** Multivariate-adjusted linear-regression models of the associations between tertiles of maternal vitamin B12 concentrations in the first (*n* = 434) and third (*n* = 353) trimester of pregnancy and Bayley Scales of Infant Development III (BSID-III) scores at 40 days after birth.

Determinants, BSID-III	First Trimester			Third Trimester		
	β	95% CI	*p*-Value	β	95% CI	*p*-Value
Motor Scale						
Vitamin B12 tertiles (0: T1, 1: T2)	2.766	0.029, 5.504	**0.048**	0.567	−2.537, 3.671	0.720
Vitamin B12 tertiles (0: T1, 1: T3)	0.918	−1.787, 3.624	0.505	0.000	−3.052, 3.053	1.000
Educational level (0: primary/secondary, 1: university)	3.011	0.344, 5.678	**0.027**	3.126	0.132, 6.119	0.041
Smoking habit (0: no, 1: yes)	5.140	1.695, 8.585	**0.004**	4.489	0.684, 8.293	0.021
Physical activity (METS/min/week) tertiles (0: T1, 1: T2)	2.901	0.170, 5.631	**0.037**	3.346	0.220, 6.472	0.036
Physical activity (METS/min/week) tertiles (0: T1, 1: T3)	3.590	0.875, 6.306	**0.010**	4.297	1.209, 7.385	0.007
	R^2^ = 0.039, F = 23, 410 = 1.59, *p* = **0.041**			R^2^ = 0.043, F = 23, 328 = 1.50, *p* = 0.068		
Fine Motor Subscale *						
Vitamin B12 tertiles (0: T1, 1: T2)	0.035	−0.008, 0.079	0.115	0.019	−0.032, 0.070	0.460
Vitamin B12 tertiles (0: T1, 1: T3)	0.003	−0.041, 0.046	0.901	−0.007	−0.057, 0.043	0.767
Educational level (0: primary/secondary, 1: university)	0.058	0.014, 0.101	**0.009**			
Smoking habit (0: no, 1: yes)	0.073	0.017, 0.129	**0.010**	0.067	0.005, 0.130	**0.035**
	R^2^ = 0.050, F = 23, 410 = 1.83, *p* = **0.013**			R^2^ = 0.050, F = 23, 328 = 1.30, *p* = 0.162		
Gross Motor Subscale						
Vitamin B12 tertiles (0: T1, 1: T2)	0.706	0.153, 1.260	**0.012**	−0.207	−0.824, 0.410	0.510
Vitamin B12 tertiles (0: T1, 1: T3)	0.279	−0.267, 0.827	0.315	−0.033	−0.640, 0.573	0.914
Physical activity (METS/min/week) tertiles (0: T1, 1: T2)	0.396	−0.166, 0.958	0.167	0.522	−0.108, 1.152	0.104
Physical activity (METS/min/week) tertiles (0: T1, 1: T3)	0.858	0.307, 1.409	**0.002**	0.923	0.310, 1.536	0.003
Vitamin B12 intake (g)	0.278	0.035, 0.522	**0.025**			
Neonatal weight–length ratio (g/m)	0.047	0.012, 0.082	**0.007**	0.053	0.014, 0.091	0.007
Birth head circumference (cm)				−0.250	−0.491, −0.009	0.041
	R^2^ = 0.047, F = 23, 410 = 1.61, *p* = **0.037**			R^2^ = 0.046, F = 23, 328 = 1.54, *p* = 0.055		
Language Scale						
Vitamin B12 tertiles (0: T1, 1: T2)	2.199	0.191, 4.207	**0.032**	−0.115	−2.358, 2.128	0.919
Vitamin B12 tertiles (0: T1, 1: T3)	1.083	−0.890, 3.056	0.281	0.809	−1.394, 3.013	0.471
Vitamin B12 intake (g)	−0.929	−1.807, −0.051	**0.038**	−1.519	−2.520, −0.518	0.003
Gestational age at birth (weeks)	0.522	0.149, 0.895	**0.006**			
Mother’s anxiety state 3rd trimester (score)				−0.166	−0.316, −0.015	0.031
	R^2^ = 0.062, F = 23, 410 = 1.58, *p* = **0.043**			R^2^ = 0.063, F = 23, 328 = 1.31, *p* = 0.158		
Receptive-Language Subscale						
Vitamin B12 tertiles (0: T1, 1: T2)	0.545	0.040, 1.050	0.034	−0.164	−0.746, 0.416	0.577
Vitamin B12 tertiles (0: T1, 1: T3)	0.056	−0.440, 0.554	0.822	0.222	−0.348, 0.794	0.444
Vitamin B12 intake (g)				−0.325	−0.584, −0.066	0.014
Gestational age at birth (weeks)	0.127	0.033, 0.221	0.008			
	R^2^ = 0.055, F = 23, 410 = 1.42, *p* = 0.094			R^2^ = 0.052, F = 23, 328 = 1.17, *p* = 0.268		
Expressive-Language Subscale						
Vitamin B12 tertiles (0: T1, 1: T2)	0.216	−0.163, 0.596	0.263	0.128	−0.282, 0.539	0.540
Vitamin B12 tertiles (0: T1, 1: T3)	0.323	−0.050, 0.697	0.090	0.057	−0.346, 0.461	0.780
Total energy intake (kcal/day)				0.000	0.000, 0.001	0.019
Vitamin B12 intake (g)				−0.199	−0.381, −0.017	0.032
Parenting Stress Index (score)	0.027	0.006, 0.048	0.010	0.024	0.001, 0.046	0.038
	R^2^ = 0.052, F = 23, 410 = 1.47, *p* = 0.074			R^2^ = 0.057, F = 23, 328 = 1.19, *p* = 0.249		
Cognitive Scale *						
Vitamin B12 tertiles (0: T1, 1: T2)	0.267	0.005, 0.048	**0.017**	0.007	−0.015, 0.029	0.538
Vitamin B12 tertiles (0: T1, 1: T3)	0.003	−0.019, 0.024	0.798	0.011	−0.011, 0.033	0.315
Educational level (0: primary/secondary, 1: university)	0.028	0.0068, 0.049	**0.010**			
Smoking habit (0: no, 1: yes)	0.032	0.005, 0.050	**0.021**			
Gestational age at birth (weeks)				0.005	0.001, 0.009	0.022
	R^2^ = 0.047, F = 23, 410 = 1.56, *p* = **0.048**			R^2^ = 0.057, F = 23, 328 = 0.98, *p* = 0.489		

Linear-regression models were used to calculate the β coefficient (β) and 95% confidence interval (95% CI). Models were performed adjusting for the following variables: vitamin B12 tertiles at 1st trimester (T1 (*n* = 146), reference: <312 pg/mL (<230 pmol/L), T2 (*n* = 145): 312–408 pg/mL (230–301.1 pmol/L), and T3 (*n* = 143): ≥409 pg/mL (≥301.8 pmol/L)) and vitamin B12 tertiles at 3rd trimester (T1 (*n* = 118), reference: <232 pg/mL (<171.2 pmol/L), T2 (*n* = 118): 232–318 pg/mL (171.2–234.7 pmol/L), and T3 (*n* = 117): ≥319 pg/mL (≥235.4 pmol/L)) depending on the main exposure, maternal age (years), BMI (0: <25 kg/m^2^, 1: ≥25 kg/m^2^), gestational weight gain (kg), educational level (0: primary/secondary, 1: university), social class (low/medium, high), smoking (0: no, 1: yes), previous parity (0: no, 1: yes), physical activity (METS/min/week, tertiles), total energy intake (kcal/day), adherence to the Mediterranean diet (score), vitamin B12 intake (µg), folate intake (µg), RBC folate levels (nmol/L), Parenting Stress Index (score), mother’s anxiety state 1st trimester (score), mother’s anxiety state 3rd trimester (score), sex of child (0: male, 1: female), gestational age at birth (weeks), type of lactation (0: breastfeeding, 1: formula/mixed), neonatal weight–length ratio (g/m), and birth head circumference (cm). * Fine motor and cognitive scales were not normally distributed and therefore log-transformed; this table presents log-transformed data. *p*-values in bold type are statistically significant.

## Data Availability

The datasets generated and/or analyzed during the current study are not publicly available due to subject confidentiality but are available from the corresponding author on reasonable request.

## Supplementary Materials

The following supporting information can be downloaded at: https://www.mdpi.com/article/10.3390/nu15061529/s1, Table S1: Maternal characteristics (sociodemographic data, health habits, nutrition, and psychological aspects) of participants included and not included in the analysis.
