# Supplementary material for: Maternal Vitamin B12 Status during Pregnancy and Early Infant Neurodevelopment: The ECLIPSES Study
Source: Nutrients. 2023 Mar 22;15(6):1529. doi: 10.3390/nu15061529 (PMC10051123; doi:10.3390/nu15061529)
Supplement: Supplementary file 1 [file nutrients-15-01529-s001.zip › nutrients-2221999-supplementary.pdf]

nutrients

MDPI

Article

Maternal Vitamin B12 Status during Pregnancy and Early Infant Neurodevelopment: the ECLIPSES Study

Josué Cruz-Rodríguez [1], Andrés Díaz-López [1,2], Josefa Canals-Sans [1,2,3] and Victoria Arija [1,2,4,5,*]

Table S1. Maternal characteristics (sociodemographic data, health habits, nutrition, and psychological aspects) of participants included and not included in the analysis.

Maternal characteristics	Included (n = 434)	Not included (n = 357)	p-value
Age (years) #	30.8 ± 5.0	29.4 ± 5.0	<0.001
BMI initial (kg/m²) #	24.8 ± 4.3	25.3 ± 4.7	0.142
Gestational weight gain (kg) #	10.3 ± 3.6	10.4 ± 3.2	0.978
Educational level, n (%)			
Low (primary/secondary)	278 (64.1)	286 (80.1)	<0.001
High (university)	156 (35.9)	71 (19.9)	
Social class, n (%)			
Low/medium	354 (81.6)	304 (85.2)	0.179
High	80 (18.4)	53 (14.8)	
Smoking during pregnancy, n (%)			
No	371 (85.5)	289 (80.9)	0.090
Yes	63 (14.5)	68 (19.1)	
Alcohol consumption during pregnancy, n (%)			
No	363 (86.4)	274 (88.4)	0.433
Yes	57 (13.6)	36 (11.6)	
Physical activity during pregnancy (METs/min/week) #	2362.8 ± 2473.7	2607.3 ± 3170.3	0.261
MedDiet during pregnancy (score) #	9.7 ± 2.1	9.5 ± 2.5	0.545
Energy intake during pregnancy (kcal) #	2087.1 ± 470.3	2184.9 ± 526.0	0.012
Vitamin B12 intake during pregnancy (µg) #	4.3 ± 1.2	4.5 ± 1.3	0.135
Folate intake during pregnancy (µg) #	199.9 ± 59.3	200.2 ± 60.9	0.961
Previous parity, n (%)			
No	190 (43.8)		
Yes	244 (56.)		
Parenting Stress Index #	50.6 ± 7.9	51.6 ± 6.6	0.135
Mother anxiety state 1st trimester (score) #	17.3 ± 8.5	18.7 ± 9.2	0.081
Mother anxiety state 3rd trimester (score) #	19.2 ± 8.7	19.0 ± 8.4	0.837
Vitamin B12 levels 1st trimester (pg/mL) #	374.2 ± 127.7	359.9 ± 124.1	0.159
Vitamin B12 levels 3rd trimester (pg/mL) # +	305.2 ± 138.0	276.1 ± 116.4	0.100
RBC Folate levels 1st trimester (nmol/L)	570.4 ± 207.3	544.3 ± 196.6	0.137

Values are expressed as a mean ± SD (standard deviation) # or n=number (%). Abbreviations: BMI, body mass index; METs, metabolic equivalent of task, MedDiet, Adherence to the Mediterranean diet; RBC folate, Red blood cell folate. Missing value: Physical activity during pregnancy [n = 90(12.8%)]; MedDiet [n = 61(8.3%)]; Energy intake during pregnancy [n = 61(8.3%)]; Vitamin B12 intake during pregnancy [n = 61(8.3%)]; Folate intake during pregnancy [n = 61(8.3%)]; Parenting Stress Index, [n = 191(31.8%)]; Mother anxiety state 1stT, [n=123(18.4%)]; Mother anxiety state 3rdT [n = 314(65.8%)]; Vitamin B12 levels 1stT [n = 119(17.7%)], Vitamin B12 levels 3rdT [n = 365(85.6%)], RBC folate levels, [n = 229(40.7%)].
